# Multiplex Gene Editing and Effect Analysis of Yield, Fragrance, and Blast Resistance Genes in Rice

**DOI:** 10.3390/genes17010077

**Published:** 2026-01-09

**Authors:** Shuhui Guan, Yingchun Han, Jingwen Zhang, Yanxiu Du, Zhen Chen, Chunbo Miao, Junzhou Li

**Affiliations:** Henan Provincial Key Laboratory of Molecular Breeding and Efficient Production of Rice, College of Agronomy, Henan Agricultural University, Zhengzhou 450046, China; guan2001st@126.com (S.G.); yingchun1002@163.com (Y.H.); m15670967790@163.com (J.Z.); duyanxiu@henau.edu.cn (Y.D.); chenzhen-win2009@163.com (Z.C.)

**Keywords:** rice, CRISPR/Cas9, *GS3*, *Gn1a*, fragrance, *Pi21*

## Abstract

Background: The coordinated improvement of yield, quality and resistance is a primary goal in rice breeding. Gene editing technology is a novel method for precise multiplex gene improvement. Methods: In this study, we constructed a multiplex CRISPR/Cas9 vector targeting yield-related genes (*GS3*, *OsPIL15*, *Gn1a*), fragrance gene (*OsBADH2*) and rice blast resistance gene (*Pi21*) to pyramid traits for enhanced yield, quality, and disease resistance in rice. A tRNA-assisted CRISPR/Cas9 multiplex gene editing vector, M601-*OsPIL15*/*GS3*/*Gn1a*/*OsBADH2*/*Pi21*-gRNA, was constructed. Genetic transformation was performed using the *Agrobacterium*-mediated method with the japonica rice variety Xin Dao 53 as the recipient. Mutation editing efficiency was detected in T_0_ transgenic plants. Grain length, grain number per panicle, thousand-grain weight, 2-acetyl-1-pyrroline (2-AP) content, and rice blast resistance of homozygous lines were measured in the T_3_ generations. Results: Effectively edited plants were obtained in the T_0_ generation. The simultaneous editing efficiency for all five genes reached 9.38%. The individual gene editing efficiencies for *Pi21*, *GS3*, *OsBADH2*, *Gn1a*, and *OsPIL15* were 78%, 63%, 56%, 54%, and 13%, respectively. Five five-gene homozygous edited lines with two genotypes were selected in the T_2_ generation. In the T_3_ generation, compared with the wild-type (WT), the edited homozygous lines showed increased grain number per panicle (14.60–25.61%), increased grain length (7.39–11.16%)**,** increased grain length–width ratio (8.37–13.02%), increased thousand-grain weight (3.79–9.15%), a 42–64 folds increase in the fragrant substance 2-AP content, and significantly enhanced rice blast resistance. Meanwhile, there were no significant changes in other agronomic traits. Conclusions: CRISPR/Cas9-mediated multiplex gene editing technology enabled the simultaneous editing of genes related to rice yield, quality, and disease resistance. This provides an effective approach for obtaining new japonica rice germplasm with blast resistance, long grains, and fragrance.

## 1. Introduction

Rice (*Oryza sativa* L.) is one of the world’s most important food crops. Global rice cultivation area exceeds 160 million hectares, supplying staple food for 3.5 billion people. However, population growth, the reduction in arable land, and intensifying climate change are presenting rice production with unprecedented challenges. Addressing biotic and abiotic stresses to ensure rice yield and meet food demand is currently crucial, as is improving rice quality to satisfy demand for high-quality agricultural products.

Rice yield is a complex trait influenced by various factors and genes [1]. Key factors affecting rice yield formation include grain number per panicle and grain weight, while grain size is a critical trait that not only affects grain weight, but also affects appearance quality [2]. Several quantitative trait loci (QTLs) that regulate grain size have been reported, such as *GW2* [3], *CLG1* [4], *DEP1* [5], *MOG1* [6] and *OsMKK4* [7,8]. Loss-of-function mutations in the grain size regulatory genes *GS3* and *GW5* can significantly increase the grain length–width ratio [9,10]. *OsPIL15* knockout lines can increase grain size and weight [11]. The *Gn1a* gene primarily regulates grain number per panicle in rice. Lower expression of *Gn1a* leads to cytokinin accumulation, which promotes the division and differentiation of panicle cells, thereby increasing grain number per panicle [12]. Fragrance has become an important quality indicator of high-quality rice and a key trait for achieving high added value. The aromatic character is primarily due to the accumulation of 2-acetyl-1-pyrrolidine (2-AP), the synthesis of which is regulated by the *OsBADH2* gene [13]. The enzyme activity encoded by *OsBADH2* decreases or disappears in mutant plants, leading to the accumulation of 2-AP in rice and affecting its fragrance [14]. Thus, rice with slender grains and rich aroma is highly favored by consumers. However, rice also faces different biotic and abiotic stresses during its growth, which is also an important focus in the study of rice yield and quality regulation mechanisms. The biotic stress factors often severely restrict rice yield potential. Among them, rice blast disease causes 10–30% yield loss globally every year. Rice blast is a disease caused by filamentous fungi that can occur throughout the entire growth period of rice, causing significant economic losses if severe. The rice blast resistance gene was identified within the QTL *Pi21*, and the loss of function of the *Pi21* gene in rice varieties susceptible to rice blast endows most rice varieties with disease resistance [15]. Key genes or QTLs controlling yield, quality, and resistance are usually scattered across different germplasm resources. Although traditional breeding methods can gradually accumulate some favorable gene alleles, they cannot simultaneously improve multiple complex traits due to the difficulty in breaking unfavorable gene linkage relationships and longer breeding cycles. Gene editing technology can accurately and efficiently integrate multiple favorable allelic variations, thereby improving rice resistance, yield and quality synergistically.

Gene editing is the process of modifying specific targets in an organism’s genome. CRISPR/Cas (Clustered Regularly Interspaced Short Palindromic Repeats/CRISPR-associated) technology is an effective gene editing tool that enables RNA-guided DNA recognition and editing. Conventional crossbreeding struggles to fix multiple beneficial recessive traits simultaneously, whereas CRISPR-mediated targeted editing provides a solution [16,17]. The introduction of the CRISPR/Cas system has significantly lowered the barrier to gene editing, enabling scientists to perform targeted genome modifications more rapidly and precisely [18,19]. This technology has been widely applied in crops, such as rice [20,21], maize [22,23], wheat [24,25] and soybeans [26,27]. Key agronomic traits, such as crop yield, disease resistance, stress tolerance, and nutritional quality, are controlled by different genes. Multiplex gene editing can stack beneficial alleles and remove yield-limiting factors, playing a crucial role in crop genetic improvement. Editing three genes, *OsPIN5b* (a panicle length gene), *GS3* (a grain weight gene), and *OsMYB30* (a cold tolerance gene), simultaneously using the CRISPR/Cas9 system resulted in high-yield, cold-tolerant mutants, and the T_2_ generations of mutants exhibited higher yield and better cold tolerance [28]. Knocking out homologs of the rice *salicylic acid 5-hydroxylase* gene (*OsS5H*) using CRISPR/Cas9-mediated gene editing generated an *oss5h1oss5h2oss5h3* triple mutant that enhanced resistance to pathogens by accumulating salicylic acid and activating defense genes [29]. In summary, CRISPR/Cas9 system, with its characteristics of precision, efficiency, and multiple editing capabilities, is driving crop breeding into a new era. It can effectively overcome the bottlenecks of long cycle and low efficiency in the pyramid formation of multiple excellent traits in traditional breeding, but also provide an effective technical approach for the coordinated improvement of various agronomic traits, such as yield, resistance, and quality.

In this study, the japonica rice cultivar Xindao53 was used as the recipient material for genetic transformation. It has low resistance to rice blast, short and round grains, and no fragrance. Using CRISPR/Cas9 technology, we constructed a multiplex gene editing vector simultaneously targeting the grain length gene *GS3*, grain size gene *OsPIL15*, grain number per panicle gene *Gn1a*, fragrance gene *OsBADH2*, and rice blast resistance gene *Pi21*. The vector was introduced into the cultivar Xindao53 to generate new rice germplasm with rice blast resistance, high yield, slender grains, and fragrance. This study provided genetic resources for breeding blast-resistant, long-grain, fragrant japonica rice varieties.

## 2. Materials and Methods

### 2.1. Experimental Materials and Cultivation Methods

The conventional japonica rice variety Xindao53 (XD53), which was bred by Xinxiang Academy of Agricultural Sciences, was used as the transgenic receptor material. XD53 is suitable for planting as wheat stubble rice in the Huang-Huai rice region of China. It possesses strong tillering ability, moderate plant type, and good ripening appearance. However, it susceptible to rice blast, the grains are short and round, and the rice has no fragrance. Field trials were conducted in Xinxiang City, Henan Province, China (35°21′ N, 113°50′ E). The soil type was alluvial paddy soil. Wild-type and T_3_ gene edited progeny lines were sown on 7 May 2025. Seedlings at the 40-day stage (seedling phase) were transplanted into the paddy field and harvested on 1 November 2025. Each line was planted in two rows, with row lengths of 1.5 m, plant spacing of 15 cm, and inter-row spacing of 30 cm. Fertilization regimen: Basal fertilizer was 750 kg/ha compound fertilizer (15-15-15, N-P_2_O_5_-K_2_O, Henan Xinlianxin Chemicals Group Co., Ltd., Xinxiang, China). Tillering fertilizer: 150 kg/ha urea (46% N, Henan Xinlianxin Chemicals Group Co., Ltd.). Panicle fertilizer: 75 kg/ha each of urea and potassium chloride (60% K_2_O, Henan Xinlianxin Chemicals Group Co., Ltd.). Irrigation management: Maintain a shallow water layer of 3–5 cm during transplanting and tillering stages. Drain fields during late tillering to control excessive tillering. Maintain shallow water during heading. Alternate between dry and wet irrigation during grain filling.

### 2.2. Construction of CRISPR/Cas9 Expression Vector

**Figure 1:**
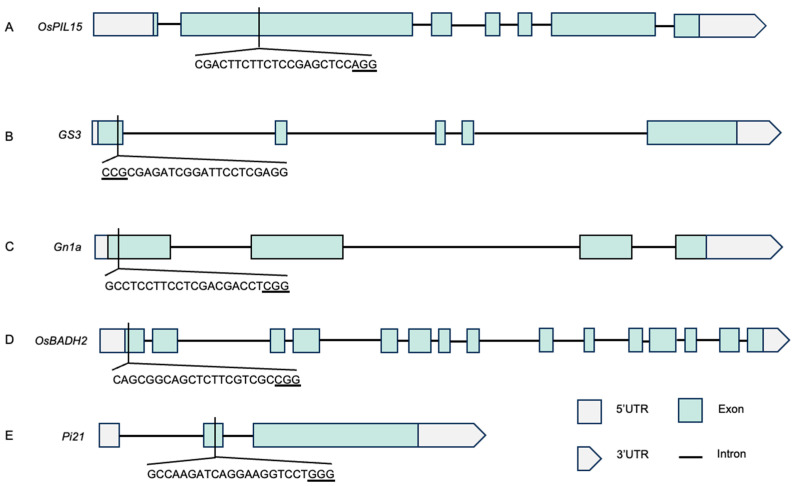
The target sites of *OsPIL15*, *GS3*, *Gn1a*, *OsBADH2*, and *Pi21*. (**A**–**E**) Schematic diagram of the gene structure and target site of *OsPIL15* (**A**), *GS3* (**B**), *Gn1a* (**C**), *OsBADH2* (**D**) and *Pi21* (**E**).

**Figure 2:**
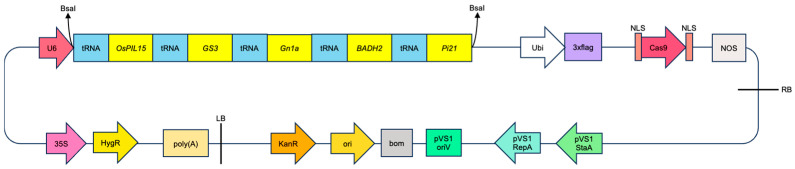
The vector construction of M601-*OsPIL15/GS3/Gn1a/OsBADH2/Pi21*-gRNA. The construct harbors a U6-driven tRNA-gRNA array, with each gRNA cassette (yellow) directed against a distinct target gene (*OsPIL15*, *GS3*, *Gn1a*, *OsBADH2*, or *Pi21*).

The vector used in the experiment was the M601 vector. The CDS sequences of *OsPIL15* (Os01g0286100), *GS3* (Os03g0407400), *Gn1a* (Os01g0197700), *OsBADH2* (Os08g0424500) and *Pi21* (Os04g0401000) were obtained from the NCBI website. The target sequence was selected as the first or second exon. The detailed positions and sequence information were shown in Figure 1. Perform target specificity assessment using the BLAT tool on the RAPDB website (https://rapdb.dna.affrc.go.jp, accessed on 1 June 2022). Then, the sgRNA sequences of yield-related genes (*OsPIL15*, *GS3*, and *Gn1a*), fragrance gene (*OsBADH2*), and rice blast resistance gene (*Pi21*) were tandemly connected by multiple tRNAs. The M601 vector was linearized using *Bsa*I, and the linearized vector was ligated with the tRNA-tandem sgRNA sequence using T4 DNA ligase to obtain the five-gene editing vector (Figure 2). The primers used are listed in Table S1.

### 2.3. Acquisition and Detection of Transgenic Plants

The M601-*OsPIL15*/*GS3*/*Gn1a*/*OsBADH2*/*Pi21*-gRNA vector was introduced into the callus XD53 using *Agrobacterium*-mediated transformation. Following hygromycin selection, the T_0_ regenerated plants were differentiated, acclimatized, and transplanted into the greenhouse. At the tillering stage, the DNA of leaf from T_0_ plants was extracted using the CTAB method. PCR detection was then performed using Cas9-F/R primers. Plants showing a 578 bp PCR product were identified as positive transgenic plants. The editing results of *OsPIL15*, *GS3*, *Gn1a*, *OsBADH2*, and *Pi21* genes were detected using Seq1-F/R, Seq2-F/R, Seq3-F/R, Seq4-F/R and Seq5-F/R, respectively. The editing efficiency of positive T_0_ generation seedlings was determined, and homozygous mutants were identified in the T_1_ and T_2_ generations through sequencing results. The primers are listed in Table S1.

### 2.4. Investigation of Yield-Related Traits

At maturity, sampling and surveys were conducted on field-grown wild-type and gene-edited progeny. Nine plants were randomly selected from each rice material, and the following traits were measured: panicle number per plant, panicle length, grain number per panicle, seed setting rate, grain yield per plant, and plant height. The thousand-grain weight were measured using mixed grains of each plant. Twenty fully filled grains from transgenic and wild-type plants were then chosen at random, and their length, and width were determined. The biological replication was also indicated in each section of the legend.

### 2.5. Determination of Fragrance Substance Content

Randomly select three rice plants to determine their aroma compounds. The content of fragrance substance, 2-acetyl-1-pyrrolidine (2-AP), was measured using gas chromatography-mass spectrometry (GC-MS). 2,4,6-Trimethylpyridine (TMP, Shanghai Aladdin Biochemical Technology Co., Ltd., Shanghai, China) was used as an internal standard during measurement process. GC conditions: HP-5MS column (J&W, New Brighton, MN, USA), inlet temperature 240 °C; temperature program: hold at 50 °C for 1 min; then ramp to 160 °C at 10 °C/min, hold for 1 min; finally ramp to 300 °C at 40 °C/min. MS conditions: electron impact ion source, ion source temperature 250 °C; full scan mode, scan range *m*/*z* 35–500. Multiple Reaction Monitoring (MRM) technology was used to generate product ions via collision-induced dissociation, and these ion signals were monitored in the third quadrupole. The molecular ion peak for 2-AP was *m*/*z* 111, with characteristic fragment ion peaks at *m*/*z* 83 and 69. The molecular ion peak for TMP was *m*/*z* 121, with characteristic fragment ion peaks at *m*/*z* 106 and 79. Qualitative analysis of 2-AP and TMP was performed using NIST library search.

### 2.6. Identification of Rice Blast Resistance

Circles of filter paper, approximately 8 cm in diameter, were placed flat inside 9 cm diameter plastic Petri dishes, and moistened with sterile water. The leaves from rice seedlings at the 4–6 leaf stage were used. When 90% of the seedling heart leaves were unfolded, a leaf segment approximately 5 cm long was cut from the middle and lower parts. Two to three wounds were made on the main vein using a dissecting needle, taking care not to penetrate the leaf. A 0.01% (*v*/*v*) Tween 20 (Beijing Solarbio Science & Technology Co., Ltd., Beijing, China) solution was sprayed onto the leaves to form a thin mist [30]. Mycelium blocks (0.5 cm × 0.5 cm) were taken from the edge of colonies cultured on an oat tomato agar medium and placed face down on the leaf wounds. The inoculated rice leaves were then incubated in darkness at 28 °C with 100% relative humidity for 30–32 h, followed by a light incubation period of 72–96 h. The symptoms on the inoculated rice leaves were observed periodically and the lesion types were recorded. Inoculation of Nipponbare (NIP) was used as the control. Strains 23-JGS-12 and 23-JGS-13 were isolated from paddy fields in Jinggangshan, China. Strain PY-DS-1-1 was isolated from paddy fields in Yuanyang, China.

### 2.7. Statistical Analysis Methods

Statistical analysis and graphical representation were performed employing GraphPad Prism software (version 10.0). Differences were assessed by one-way ANOVA.

## 3. Results

### 3.1. Analysis of Editing Efficiency of T_0_ Generation Transgenic Plants

**Figure 3:**
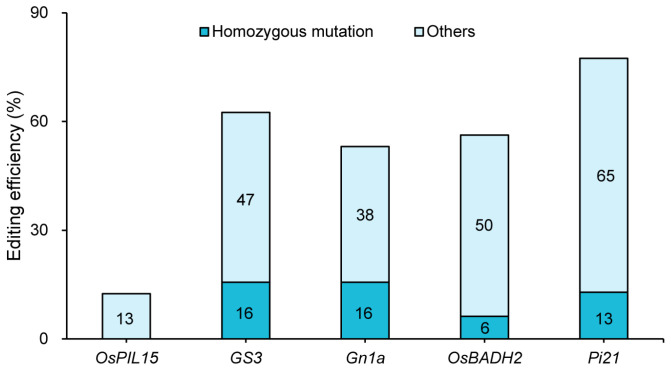
Efficiency of five-gene editing of T_0_ generation.

Based on the identification results of the edited plants in T_0_ generation, the simultaneous editing efficiency for all five genes was 9.38%. The highest single-gene editing efficiency was *Pi21* (78%), followed by *GS3* (63%), *OsBADH2* (56%), and *Gn1a* (54%), while *OsPIL15* has the lowest efficiency, only 13%. Of all plants that were successfully edited, the highest homozygous editing efficiency was for *Pi21* (12.90%), followed by *Gn1a* and *GS3* (both 15.63%), and *OsBADH2* (6.25%), while *OsPIL15* has no homozygous editing (Figure 3).

### 3.2. Screening and Identification of Homozygous Mutant Lines of Five Genes

**Figure 4:**
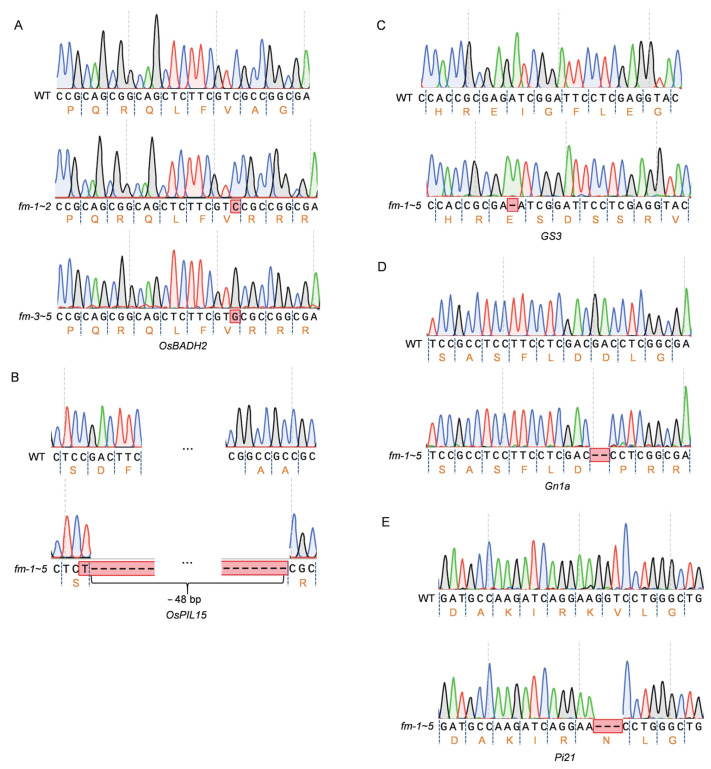
The mutant sites of homozygous edited lines. (**A**–**E**) The mutation sites of *OsBADH2* (**A**), *OsPIL15* (**B**), *GS3* (**C**), *Gn1a* (**D**), and *Pi21* (**E**) in the homozygous edited lines.

The sequences containing the target site of each individual plant in the T_2_ generation were amplified using sequencing primers, Seq1-F/R, Seq2-F/R, Seq3-F/R, Seq4-F/R and Seq5-F/R. Five lines with all 5 genes homozygous, namely *fm-1*, *fm-2*, *fm-3*, *fm-4*, and *fm-5*, were identified by sequencing analysis. And two genotypes were identified (Figure 4). The genotypic difference between *fm-1* and *fm-2* versus *fm-3*, *fm-4*, and *fm-5* was the mutation type in *OsBADH2*, which is a single-base insertion of G or C (Figure 4A). Both insertions result in premature translation termination. The other four genes showed no difference between genotypes. The mutation type of *OsPIL15* was a base insertion and deletion (+T; −48 bp) (Figure 4B); *GS3* exhibits a single-base deletion (−G) (Figure 4C), *Gn1a* a double-base deletion (−GA) (Figure 4D), and *Pi21* a triple-base deletion (−GGT) (Figure 4E). The mutation types of *OsPIL15*, *GS3*, and *Gn1a* all resulted in premature translation termination, whereas the protein sequence of *Pi21* lacked one amino acid (KV-N) compared to that of wild-type (Figure 4).

### 3.3. Analysis of Yield Traits of Homozygous Edited Lines

**Figure 5:**
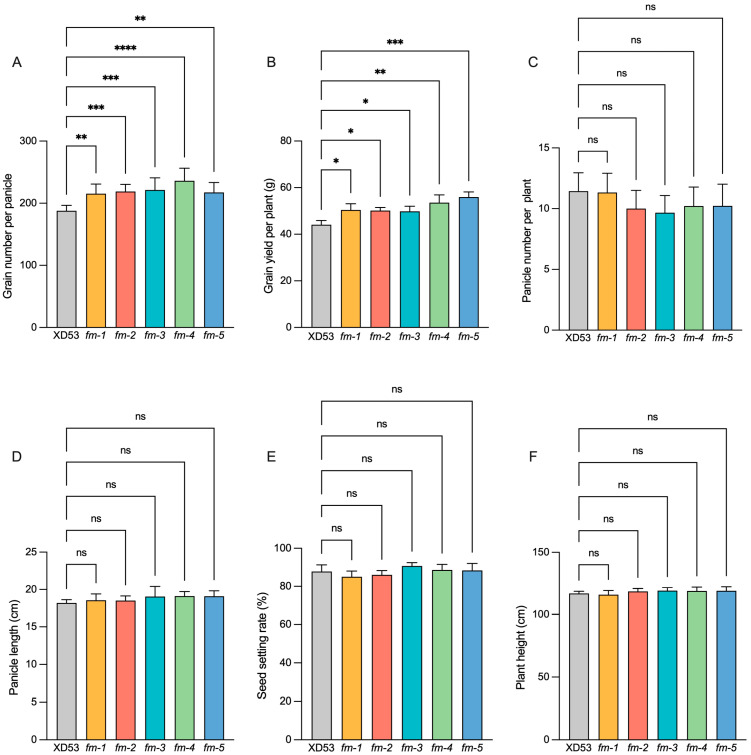
Performance of key agronomic traits in wild-type and edited lines. (**A**) Grain number per panicle. (**B**) Grain yield per plant. (**C**) Panicle number per plant. (**D**) Panicle length. (**E**) Seed setting rate. (**F**) Plant height. ((**A**,**D**,**E**): *n* = 3 with at least 3 panicles used per group, (**B**): *n* = 6, (**C**,**F**): *n* = 9) Data represent means ± s.d. * *p* ≤ 0.05, ** *p* ≤ 0.01, *** *p* ≤ 0.001, **** *p* ≤ 0.0001 (one-way ANOVA). ns, not significant.

**Figure 6:**
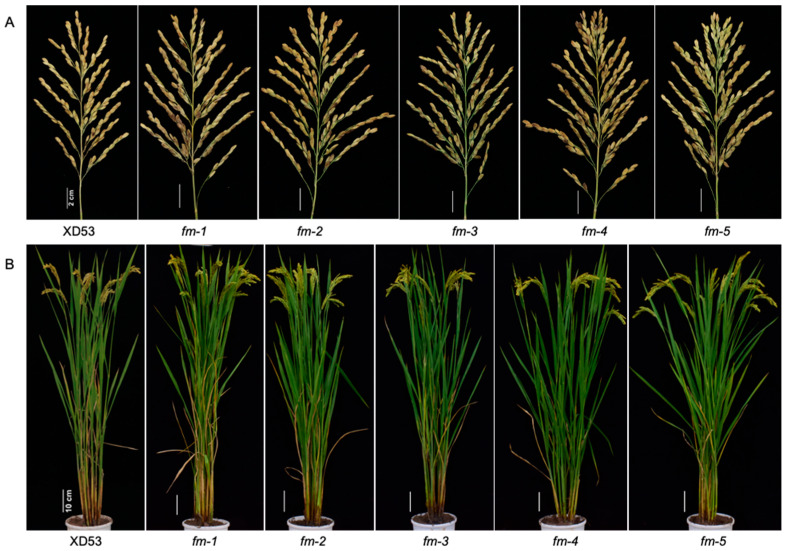
Phenotypes of panicles and whole plants in wild-type and edited lines. (**A**) Morphology of the panicles of wild-type and edited lines. Scale bar: 2 cm. (**B**) Performances of whole plant of wild-type and edited lines. Scale bar: 10 cm.

The yield traits of five five-gene homozygous mutant lines were measured. Compared to wild-type, the grain number per panicle in the five homozygous lines significantly increased by 14.60%, 16.44%, 17.80%, 25.61%, and 15.67%, respectively (Figure 5A and Figure 6A). The grain yield per plant in the five homozygous lines also significantly increased by 14.40%, 13.79%, 13.09%, 21.48%, and 26.90%, respectively (Figure 5B). However, there were no significant differences in panicle number per plant (Figure 5C), panicle length (Figure 5D), and seed setting rate (Figure 5E). In addition, there was also no significant change in plant height of the homozygous lines (Figure 5F and Figure 6B). These results indicated that simultaneous editing of all five genes did not affect normal growth, but editing of the *Gn1a* gene significantly increased grain number per panicle and grain yield per plant in rice without altering other yield traits.

### 3.4. Analysis of Grain Phenotype of Homozygous Edited Lines

**Figure 7:**
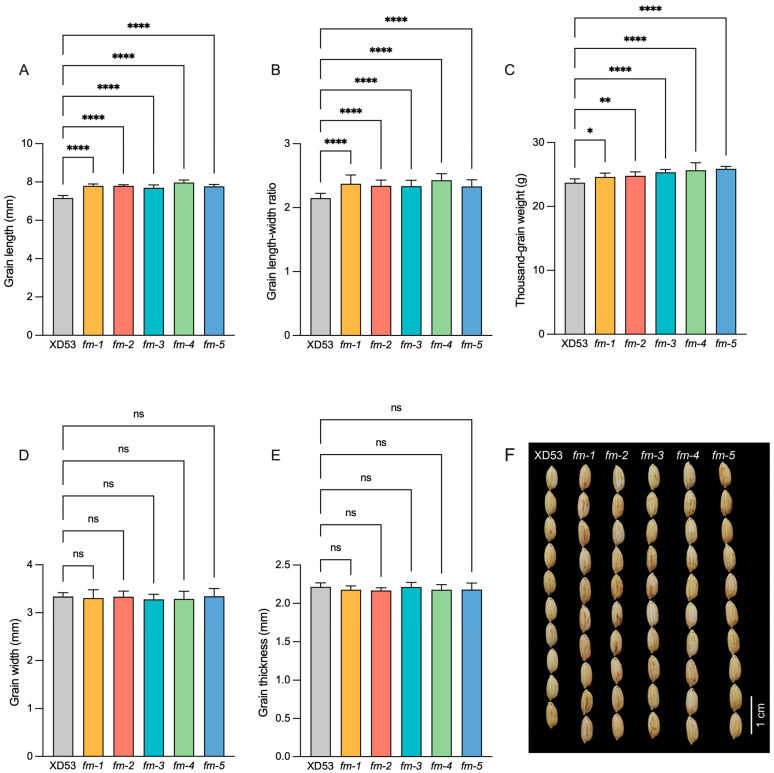
Performance of grain size in wild-type and edited lines. (**A**–**E**) The grain length (**A**), grain length–width ratio (**B),** thousand-grain weight (**C**), grain width (**D**), and grain thickness (**E**) of wild-type and edited lines. (**F**) Phenotypes of grains of wild-type and edited lines. Scale bar: 1 cm. ((**A**,**B**,**D**,**E**): *n* = 20, (**C**): *n* = 9) Data represent means ± s.d. * *p* ≤ 0.05, ** *p* ≤ 0.01, **** *p* ≤ 0.0001 (one-way ANOVA). ns, not significant.

Compared with the wild-type, the grain length and length–width ratio significantly increased in all five edited lines. The grain lengths of edited lines, *fm-1*, *fm-2*, *fm-3*, *fm-4*, and *fm-5*, were 7.79 mm, 7.79 mm, 7.70 mm, 7.97 mm, and 7.77 mm, respectively, representing increases of 7.39–11.16% compared to that of the wild-type (Figure 7A). The grain length–width ratio of the same lines was 2.37, 2.34, 2.34, 2.43, and 2.33, respectively, representing an increase of 8.37–13.02% compared to that of the wild-type (Figure 7B). The thousand-grain weight in the five homozygous lines also significantly increased by 3.79%, 4.55%, 6.79%, 8.22%, 9.15%, respectively (Figure 7C). However, there were no significant differences in grain width (Figure 7D) and grain thickness (Figure 7E). These results indicated that the editing of the *GS3* and *OsPIL15* genes significantly increased grain length, grain length–width ratio and grain weight.

### 3.5. Fragrance Analysis of Homozygous Edited Lines

**Figure 8:**
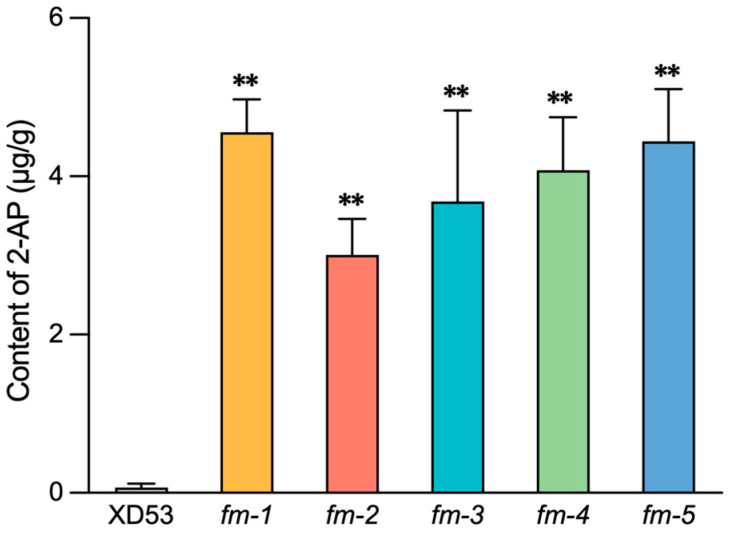
The content of 2-AP in wild-type and edited lines (*n* = 3). ** *p* ≤ 0.01 (one-way ANOVA).

The content of 2-AP in grains of wild-type and the edited lines, *fm-1*, *fm-2*, *fm-3*, *fm-4*, and *fm-5*, were analyzed. The results showed that the content of 2-AP in all five lines were significantly higher than those in the wild-type (Figure 8). The highest content of 2-AP was detected in *fm-1*, reaching 4.56 μg/g, followed by *fm-5*, *fm-4*, and *fm-3*, while *fm-2* had the lowest content, 3.01 μg/g. It showed an increase of 42–64-fold compared with the wild-type. These results indicated that the editing of the *OsBADH2* gene effectively increased the content of fragrant substances, 2-AP.

### 3.6. Rice Blast Resistance Analysis of Homozygous Edited Lines

**Figure 9:**
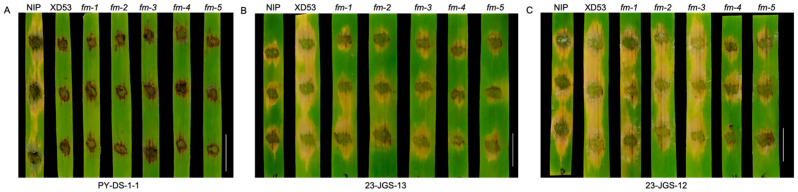
The inoculation phenotypes after inoculation with rice blast in control, wild-type and edited lines. (**A**–**C**) The inoculation phenotypes after inoculation with PY-DS-1-1 (**A**), 23-JGS-13 (**B**) and 23-JGS-12 (**C**). Scale bar: 1 cm.

Three strains were used to identify rice blast resistance. When inoculated with strain PY-DS-1-1, NIP showed susceptibility, whereas XD53 and its edited lines showed no disease symptoms (Figure 9A), indicating that XD53 was resistant to strain PY-DS-1-1, and that editing *Pi21* did not affect the resistance. When inoculated with strain 23-JGS-13, XD53 were more severely infected, with obvious lesions spreading, at 5 dpi (days post-inoculation), while the lesions on the homozygous mutant lines were significantly smaller than those of XD53, especially *fm-4* and *fm-5* (Figure 9B). Similarly to the infection results of 23-JGS-13, XD53 also showed severe infection symptoms when inoculated with 23-JGS-12, while the lesions in the homozygous mutant line were smaller than those in XD53. Among them, *fm-4* and *fm-5* showed significantly stronger resistance (Figure 9C). These results indicated that editing for the *Pi21* gene by the CRISPR/Cas9 system had enhanced resistance to certain rice blast strains at the seedling stage compared to the wild-type.

## 4. Discussion

### 4.1. Innovation and Comparison of Multiplex Gene Editing Vector Construction Strategies

The tRNA-assisted tandem multiplex gene knockout vector system was used to construct a vector that targeted *OsPIL15*, *GS3*, *Gn1a*, *OsBADH2* and *Pi21* simultaneously. The achieved co-editing of the five genes via *Agrobacterium*-mediated transformation, with a simultaneous five-gene editing efficiency of 9.38% (Figure 3). Compared with traditional multi-vector systems [28,31], this design significantly simplified the process of vector construction and avoided the risk of gene silencing associated with multiple promoters. The core innovation of this vector design lies in utilizing the self-cleaving property of tRNA to express multiple sgRNAs in tandem. It avoided issues that related to larger vector size and variation in expression efficiency caused by tandem promoters [32]. The vector used in this study solved the problems of unstable editing efficiency and higher off-target effects that exist in traditional CRISPR/Cas9 vectors [33,34]. Its characteristic was the inclusion of independent but cooperative expression units, namely the Ubi promoter (driving Cas9) and the U6 promoter (driving sgRNA) (Figure 2). The design of the vector ensured the balanced expression of the two core components, achieving efficient and high-fidelity gene editing. This method has significant advantages in vector construction complexity, conversion efficiency, and editing throughput. Furthermore, the vector using the Ubi promoter for Cas9 and the U6 promoter for sgRNA showed good expression stability and editing specificity.

### 4.2. Mechanism Analysis and Optimization Strategies for Differences in Multiplex Gene Editing Efficiency

In this study, the simultaneous five-gene editing efficiency in T_0_ plants was 9.38%, while single-gene efficiencies showed significant variation. *Pi21* has the highest efficiency (78%), while *OsPIL15* has the lowest efficiency (13%) (Figure 3). These differences in editing efficiency may be related to several factors, such as the rationality of gRNA design, GC content, the chromatin accessibility of the target region, the stability of the Cas9/sgRNA complex, and the preferences of the DNA repair mechanism [35]. For example, *OsPIL15* was located near the end of the chromosome, which could lead to low editing efficiency due to its heterochromatin state [36]. The coding region structure, exon position, and PAM sequence availability of different genes also affect editing efficiency [37]. Additionally, the U6 promoter typically requires a guanine (G) as the transcription start site, whereas the first base designed for *OsPIL15* is cytosine (C), which may also affect transcription efficiency. To improve multiplex gene editing efficiency, the following strategies can be considered: (1) Optimizing target selection by prioritizing targets located in conserved gene domains with a higher GC content to improve gRNA binding efficiency and specificity [38,39]; (2) using chromatin openers, such as histone deacetylase inhibitors, to increase chromatin accessibility in the target region and thereby enhance Cas9/sgRNA binding capacity [24]; (3) employing a “dual-target cleavage” strategy by designing two adjacent target sites within the same gene and cutting them simultaneously to significantly increase the probability of frameshift mutations [40]; and (4) using highly active Cas9 variants, such as xCas9, which have a broader PAM recognition range and higher editing precision, and are especially suitable for editing difficult-to-target genomic regions [18]. The strategy of increasing the probability of effective editing through the synergistic effect of multiple targets has been successfully applied in plant multiplex gene editing systems [29]. Therefore, one or more additional target sites may be designed within the gene to address the issue of low editing efficiency for *OsPIL15*.

### 4.3. Effects of Multiplex Gene Editing on Phenotypes

This study successfully improved grain length, grain weight, grain number per panicle, fragrance substance content, and rice blast resistance of rice through multiplex gene editing. The GS3-encoded protein inhibits cell division and elongation. In GS3-deficient rice, the longitudinal division and elongation capacity of glume and lemma cells are enhanced, increasing grain storage capacity and resulting in longer and heavier grains [9]. *Gn1a* encodes cytokinin oxidase/dehydrogenase OsCKX2, which is highly expressed in the inflorescence meristem and young panicles to degrade cytokinin. Loss of *Gn1a* function leads to increased cytokinin accumulation in the panicle, boosting grain number per panicle [12]. OsPIL15, a bHLH family transcription factor, binds the N1-box element in the *OsPUP7* promoter to influence cytokinin transport, thereby regulating cell division and grain size [11]. Simultaneously, it binds the G-box element in the *OsMIR530* promoter to activate its expression. Since OsmiR530 negatively regulates grain yield, inhibiting OsPIL15 enhances grain yield [41]. The increase in grain length and grain length–width ratio in the edited lines was primarily due to the loss-of-function of *GS3* [9,42]. However, the increase in grain length was not particularly high (7.39–11.16%), possibly due to the genetic background of XD53, which may have limited the effect of *GS3* [43]. The increase in grain number per panicle in the edited lines (14.60–25.61%) was caused by the accumulation of cytokinin in the inflorescence meristem due to the loss of function of *Gn1a*, thereby increasing the number of spikelets, consistent with the phenotype of grain number per panicle in *OsCKX2*/*Gn1a* gene-silenced lines [12,44]. The increase in thousand seed weight (3.79–9.15%) may result from the combined editing effects of GS3 and OsPIL15 [11,45]. The enhancement of fragrance in grains was due to the accumulation of 2-AP, which is caused by a mutation in the *OsBADH2* [13]. Here, the content of 2-AP in the homozygous edited lines increased by 42- to 64-fold (Figure 8). It indicated that the fragrance controlled by *OsBADH2* was a key gene for modulating rice fragrance, and editing it has a good effect on improving rice fragrance, which could be effectively applied in high-quality molecular breeding. The *Pi21* edited lines showed enhanced resistance to the rice blast strains, 23-JGS-13 and 23-JGS-12, which is similar to the previously reported results [15]. However, the edited lines showed varying resistance to different rice blast strains, and the gene editing of *Pi21* has limited effectiveness in enhancing rice blast resistance (Figure 9). It may be because the edited *Pi21* mutation only resulted in a single amino acid deletion in this study. Although this amino acid deletion affects *Pi21* protein function and partially improves rice blast resistance, it is insufficient to meet the requirements of disease resistance. More *Pi21* mutation types need to be identified in the future to screen for homozygous lines with more significant improvement in rice blast resistance.

In multiplex editing breeding studies, interactions between genes may produce synergistic or antagonistic effects. In this study, *OsBADH2* and *Pi21* regulated target traits without affecting rice growth or yield. GS3, OsPIL15, and *Gn1a*, all cytokinin-related genes, regulate cell division during different developmental stages and in various rice tissues, and negatively modulate panicle and grain development. Mutating these five genes synergistically enhanced yield traits while simultaneously improving aroma and disease resistance.

## Data Availability

All the data generated or analyzed during this study are included in this published article.

## Supplementary Materials

The following supporting information can be downloaded at: https://www.mdpi.com/article/10.3390/genes17010077/s1, Table S1: The primers used in this study.
