# Supplementary material for: Multiplex Gene Editing and Effect Analysis of Yield, Fragrance, and Blast Resistance Genes in Rice
Source: Genes (Basel). 2026 Jan 9;17(1):77. doi: 10.3390/genes17010077 (PMC12840757; doi:10.3390/genes17010077)
Supplement: Supplementary file 1 [file genes-17-00077-s001.zip › Supplemental Material.pdf]

Table S1. The primers used in this study.

Primer name	Sequence
M1-1F	aagcccgattctcttcgctgtgatg
M1-1R	cgcgctacggactagcctta
Cas9-F	GGACAACCGGGAAAAGATCG
Cas9-R	CAAACAGTGTCAGGGTCAGC
Seq1-F	GAGGAAGAAGCACCCGCAG
Seq1-R	GAACGGCATCTCCTTCGGCT
Seq2-F	GATCATCTCCATTATCGGAACTTCG
Seq2-R	TCATCAGTTCAACTTGCTTGTTTGC
Seq3-F	TCACACAGATCTCAGAGAGC
Seq3-R	GAGGTAGTCTGTCCACGACA
Seq4-F	TCCATCGTCATCGATCCATC
Seq4-R	TAGTCACCACCCTACCTTG
Seq5-F	CTGAATTCCACGGGAATTGC
Seq5-R	AACTTCCCGCGCACGATCAC
